# Intestinal fatty acid-binding protein as a biomarker for the diagnosis of strangulated intestinal obstruction: A meta-analysis

**DOI:** 10.1515/med-2021-0214

**Published:** 2021-02-02

**Authors:** Caihong Wu, Xuehe Zhu, Haipeng Ren, Fuyong Tan, Xudong Liu

**Affiliations:** Emergency Department, Affiliated Hospital of Inner Mongolia Medical University, Hohhot 010050, Inner Mongolia, China; The Medical Examination Center, Affiliated Hospital of Inner Mongolia Medical University, Hohhot 010050, Inner Mongolia, China

**Keywords:** intestinal fatty acid-binding protein, strangulated intestinal obstruction, diagnostic value, meta-analysis

## Abstract

**Objective:**

The purpose of this study was to clarify the value of intestinal fatty acid-binding protein (I-FABP) for the early diagnosis of strangulated intestinal obstruction through a meta-analysis.

**Methods:**

A search was performed on PubMed, EBSCO, the Cochrane Library, the Web of Science, EMBASE, CNKI, and WanFang for studies on the diagnosis of strangulated intestinal obstruction based on I-FABP. Endnote X9 software and the quality assessment of diagnostic accuracy studies 2 (QUADAS-2) were used to screen the studies and evaluate their quality, respectively. Meta-Disc 1.4 and Stata 15.1 software were used to perform the assessment of heterogeneity and meta-analysis.

**Result:**

A total of eight studies were included, Spearman correlation coefficient was 0.703 (*P* = 0.078), suggesting that there was no threshold effect. The pooled results of the meta-analysis were as follows: sensitivity: 0.75 (95% CI: 0.66–0.81), specificity: 0.83 (95% CI: 0.71–0.91), positive likelihood ratio (PLR): 4.35 (95% CI: 2.57–7.36), negative likelihood ratio (NLR): 0.31 (95% CI: 0.24–0.39), and diagnostic odds ratio (DOR): 14.19 (95% CI: 8.08–24.92). The area under the curve was 0.83. There was obvious heterogeneity among the studies.

**Conclusion:**

I-FABP is very valuable for the early diagnosis of strangulated intestinal obstruction and can be used to distinguish strangulated intestinal obstruction from intestinal obstruction in a timely manner, enabling accurate planning of the timing of surgery.

## 1 Introduction

Intestinal obstruction is a common cause of acute abdomen, which can be divided into simple and strangulated intestinal obstruction. There are no significant differences in the clinical manifestations of simple and strangulated intestinal obstruction in the early stage. Surgeons remove the lesion generally through laparotomy and confirm the diagnosis with histopathology [1,2]. Although abdominal multislice spiral CT is commonly used in clinical practice, it has certain limitations with regard to the identification of strangulated and simple intestinal obstruction [3,4], making it difficult to determine the surgical indications and thereby increasing the occurrence of intestinal necrosis. Although the ratio of strangulated intestinal obstruction to simple intestinal obstruction is approximately 1:9, the incidences of complications of the former, such as massive intestinal necrosis, septic shock, and even death, are high [5,6]. Published studies have indicated that the mortality rate due to strangulated intestinal obstruction is 2–10 times that due to simple intestinal obstruction [7]. Therefore, serological indicators with high sensitivity and specificity are urgently needed in clinical practice to enable the early diagnosis of strangulated intestinal obstruction.

Intestinal fatty acid-binding protein (I-FABP) is a low molecular weight (approximately 15 kDa) cytoplasmic protein [8,9]. In 1972, Ockner et al. discovered FABP. The FABP can be divided into nine types of proteins that are named according to the different organs in which they are found. Among the FABP family of proteins, I-FABP [10,11,12,13] accounts for 2% of the cytoplasmic proteins in the intestinal cells of the small intestinal mucosa, and it plays a role in the absorption and transportation of long-chain fatty acids. In recent years, many studies have reported that I-FABP also has predictive value for intestinal ischemia. When the strangulation of intestinal obstruction occurs, I-FABP is released quickly, and an increase in I-FABP can be detected in a blood test. Many researchers have studied the diagnostic value of I-FABP for strangulated intestinal obstruction, but because of the small sample size in any given individual study, the different research designs, hospital equipment, and the severity of obstruction, there is no unified conclusion drawn about the value of I-FABP for the diagnosis of intestinal obstruction. This study was a meta-analysis of the studies on the diagnostic value of I-FABP for strangulated intestinal obstruction that was performed to provide a reasonable basis for its use in clinical practice.

## 2 Materials and methods

### 2.1 Study selection

PubMed, EBSCO, the Cochrane Library, the Web of Science, EMBASE, CNKI, WanFang, and other literature databases were searched for studies on the diagnosis of strangulated intestinal obstruction based on I-FABP. The retrieval time was from 01 January 2010 to 31 December 2019. The search terms included medical subject headings, free words, and the set operator “AND” in the following three combinations: (1) “Intestinal Obstruction” OR “Intestinal necrosis” OR “Intestinal ischemia” OR “bowel obstruction” OR “colonic obstruction” OR “gut obstruction” OR “Obstruction, Intestinal”; (2) “Fatty Acid-Binding Proteins” OR “FABP” OR “FAB” OR “I-FABP” OR “Intestinal Fatty Acid-Binding Protein” OR “Enteric fatty acid binding protein” OR “Fatty Acid-Binding Proteins, Intestinal-Specific;” and (3) “sensitiv*” OR “sensitivity and specificity” OR “predictive and value*” OR “predictive value of tests” OR “accuracy*”. Since this study involved published papers, it did not need approval from the Medical Ethics Society.

### 2.2 Study inclusion and exclusion criteria

Inclusion criteria include the following. (1) Patients with intestinal obstruction who were diagnosed based on intraoperative findings, pathology, or imaging. (2) An original study of the use of I-FABP for the diagnosis of strangulated intestinal obstruction. (3) The number of true positive (TP), false positive (FP), false negative (FN), and true negative (TN) could be directly or indirectly extracted from the study.

Exclusion criteria include the following. (1) The sensitivity and specificity of I-FABP were not reported in the original study. (2) The study was unpublished. (3) There were fewer than 35 cases in the study. (4) The full-text article was unavailable or the data were incomplete. (5) The study was a review, systematic review, or animal experiment.

### 2.3 Data extraction and study quality evaluation

Based on the inclusion and exclusion criteria, two researchers used Endnote X9 software to screen the initially identified literature and select the appropriate studies for inclusion. Finally, the author, year of publication, country of publication, average age, sample size, TP, FP, FN, TN, cutoff value, and reference standard were extracted from each study after the full text was read. Two researchers applied quality assessment of diagnostic accuracy studies 2 (QUADAS-2) to evaluate the quality of the included studies. QUADAS-2 consists of 11 questions, with one point possible for each question, yielding a maximum possible score of 11 points. For each question on QUADAS-2, the researchers answered “yes,” “no,” or “unclear”. If the two researchers disagreed, a third person was consulted.

### 2.4 Statistical analysis

Meta-Disc 1.4 and Stata 15.1 software were used for the meta-analysis. The Q test and I-square test were used to evaluate the heterogeneity. If the I-square statistic was less than 30%, and *P* was more than 0.1, then there was no heterogeneity. If the I-square statistic was from 30 to 50%, then there was moderate heterogeneity. If the I-square statistic was more than 50%, and *P* was less than 0.1, then there was obvious heterogeneity. The presence of the threshold effect was assessed by calculating the Spearman correlation coefficient of logit (true positive rate [TPR]) and logit (false positive rate [FPR]). The sensitivity, specificity, positive likelihood ratio (PLR), negative likelihood ratio (NLR), and diagnostic odds ratio (DOR) were determined with a bivariate mixed-effects model, and a receiver operating characteristic (ROC) curve was drawn to calculate the accuracy of the diagnosis based on the area under the curve (AUC). Deeks’ funnel plot asymmetry test was used to evaluate publication bias, and a sensitivity analysis was conducted. The difference was considered statistically significant when *P* was < 0.05.

## 3 Results

### 3.1 General characteristics and quality evaluation of the included studies

**Figure 1:**
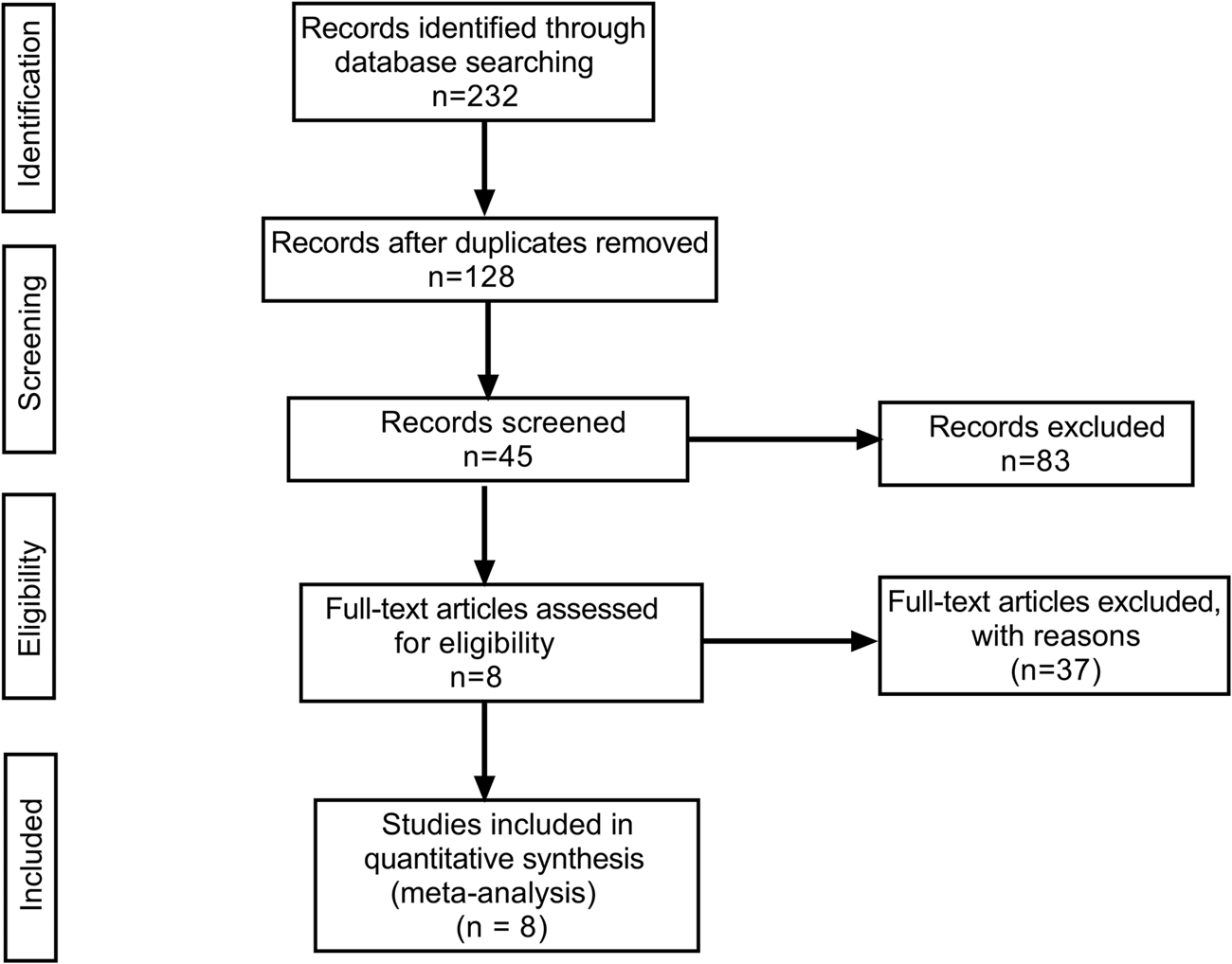
The flow chart of study inclusion in the meta-analysis.

After the preliminary retrieval of 232 studies from the aforementioned databases and screening with Endnote X9 software, eight studies were found that met the inclusion criteria, including a total of 583 patients with intestinal obstruction. The flow chart of study inclusion in the meta-analysis is shown in Figure 1. The general characteristics of the included studies are shown in Table 1. The quality evaluation of the included studies is shown in Table 2.

**Table 1:** General characteristics of the included studies

Authors	Year	Country	Average age	Sample size	TP	FP	FN	TN	Cutoff value (ng/mL)	Reference standard	I-FABP methods of detection
Kanda et al. [14]	2011	Japan	56.4	109	25	35	5	44	3.1	Surgery, imaging, and clinical diagnosis	ELISA
Yang and Qin [15]	2011	China	49.49	47	20	2	8	17	0.218	Surgery, imaging, and clinical diagnosis	ELISA
Sakamoto et al. [16]	2013	Japan	67	37	7	2	3	25	7.2	Surgery, imaging, and clinical diagnosis	ELISA
Zheng et al. [17]	2014	China	56.2	128	42	25	11	50	84.29	Surgery, imaging, and clinical diagnosis	ELISA
Kittaka et al. [18]	2014	Japan	79	37	15	1	6	15	6.5	Surgery, imaging, and clinical diagnosis	ELISA
Lu [19]	2017	China	50.7	120	41	6	17	56	0.218	Surgery, imaging, and clinical diagnosis	ELISA
Ma [20]	2018	China	58.5	40	19	6	1	14	0.3036	Surgery, imaging, and clinical diagnosis	ELISA
Hu et al. [21]	2019	China	6.53	65	21	3	15	26	24.01	Surgery, imaging, and clinical diagnosis	ELISA

**Table 2:** Quality evaluation of the included studies

Authors	Year	1	2	3	4	5	6	7	8	9	10	11	score
Kanda et al. [14]	2011	Yes	Yes	Yes	Yes	No	Yes	Yes	Yes	Yes	No	Yes	9
Yang and Qin [15]	2011	Yes	Yes	Yes	No	No	Yes	Yes	Yes	Yes	No	Yes	8
Sakamoto et al. [16]	2013	Yes	Yes	Yes	Yes	No	Yes	Yes	Yes	Yes	No	Yes	9
Zheng et al. [17]	2014	Yes	Yes	Yes	Unclear	No	Yes	Yes	Yes	Yes	No	Yes	8
Kittaka et al. [18]	2014	Yes	Yes	Yes	Yes	No	Yes	Yes	Yes	Yes	Yes	Yes	10
Lu [19]	2017	Yes	Yes	Yes	Yes	No	Yes	Yes	Yes	Yes	No	Yes	9
Ma [20]	2018	Yes	Yes	Yes	Yes	Yes	Yes	Unclear	No	Yes	No	Yes	8
Hu et al. [21]	2019	Yes	Yes	Yes	Yes	No	Yes	Unclear	No	Yes	No	Yes	7

The questions of QUADAS-2: (1) was a consecutive or random sample of patients enrolled? (2) Was a case–control design avoided? (3) Did they avoid inappropriate exclusion? (4) Where the index test results interpreted without knowledge of the results of the reference standard? (5) If a threshold was used, was it pre-specified? (6) Are the reference standards likely to correctly classify the target condition? (7) Were the reference standard results interpreted without knowledge of the results of the index test? (8) Was there an appropriate interval between index test and reference standard? (9) Did all patients accept the reference standard? (10) Did all patients receive the same reference standard? (11) Were all patients included in the analysis?

### 3.2 Pooled sensitivity and specificity

**Figure 2:**
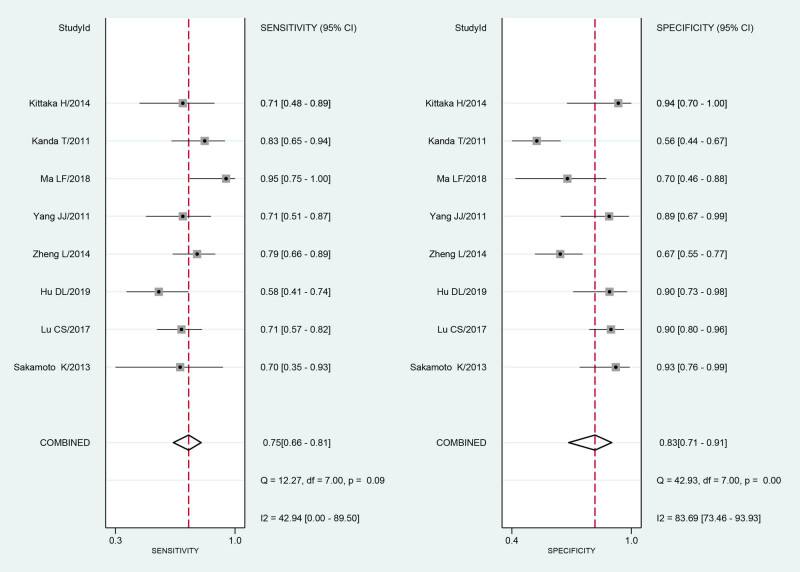
The forest plot of the diagnostic sensitivity and specificity for serum I-FABP in diagnosis of strangulated intestinal obstruction.

Based on the Q test and the I-square test, there was statistically significant heterogeneity in the pooled sensitivity (*I*² = 42.94, *P* = 0.09). There was obvious heterogeneity in the pooled specificity (*I*² = 83.69, *P* = 0.00). Therefore, a bivariate mixed-effects model was used for the analysis. The pooled sensitivity of I-FABP for the diagnosis of strangulated intestinal obstruction was 0.75 (95% CI: 0.66–0.81), and the pooled specificity was 0.83 (95% CI: 0.71–0.91) (Figure 2).

### 3.3 Pooled PLR and NLR

**Figure 3:**
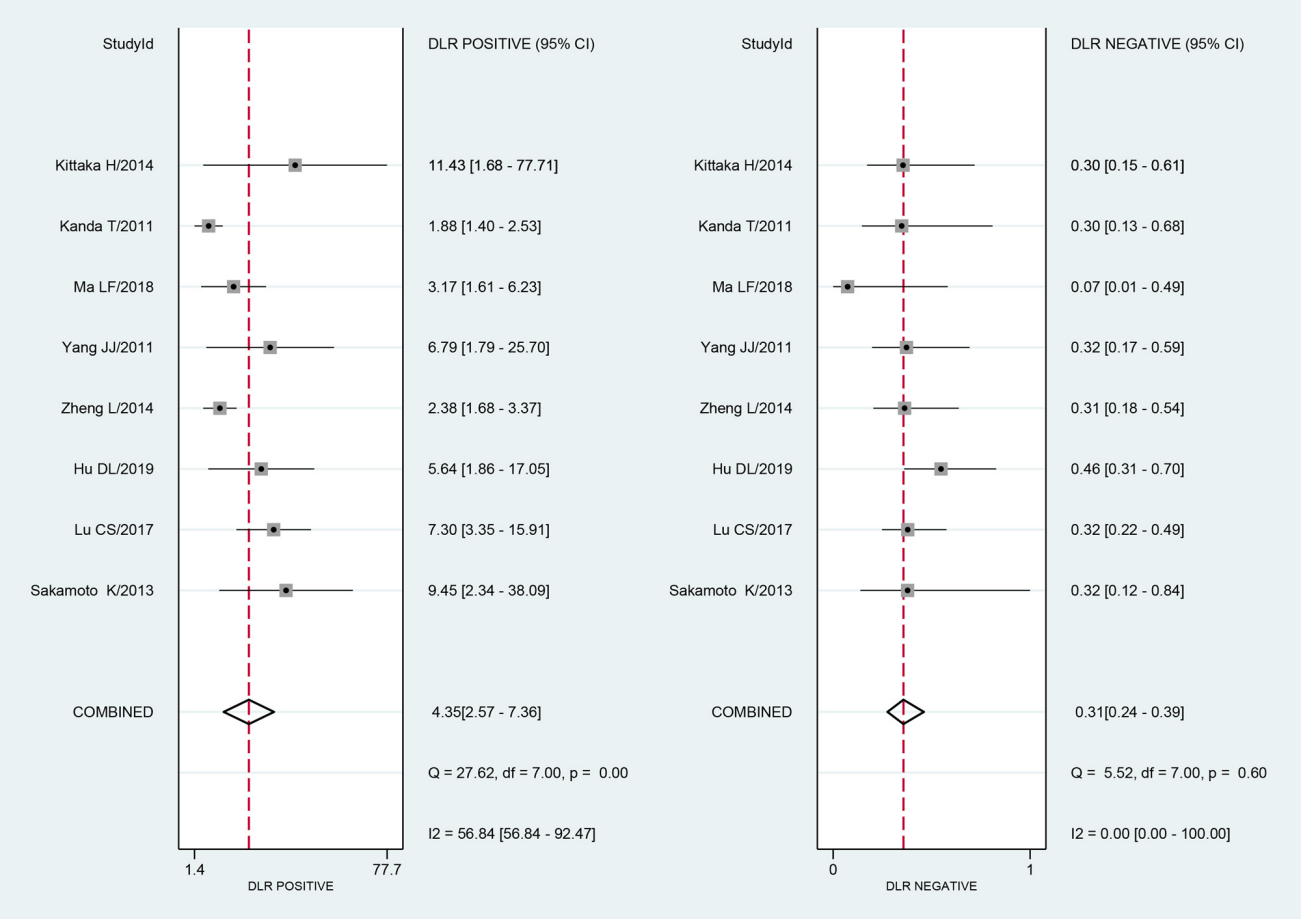
The forest plot of the diagnostic PLR and NLR for serum I-FABP in diagnosis of strangulated intestinal obstruction.

There was statistical heterogeneity in the pooled PLR (*I*² = 56.84, *P* = 0.00) and NLR (*I*² = 0.00, *P* = 0.60). A bivariate mixed-effects model was used for the analysis. The pooled PLR was 4.35 (95% CI: 2.57–7.36), and the pooled NLR was 0.31 (95% CI: 0.24–0.39) (Figure 3).

### 3.4 Pooled DOR

**Figure 4:**
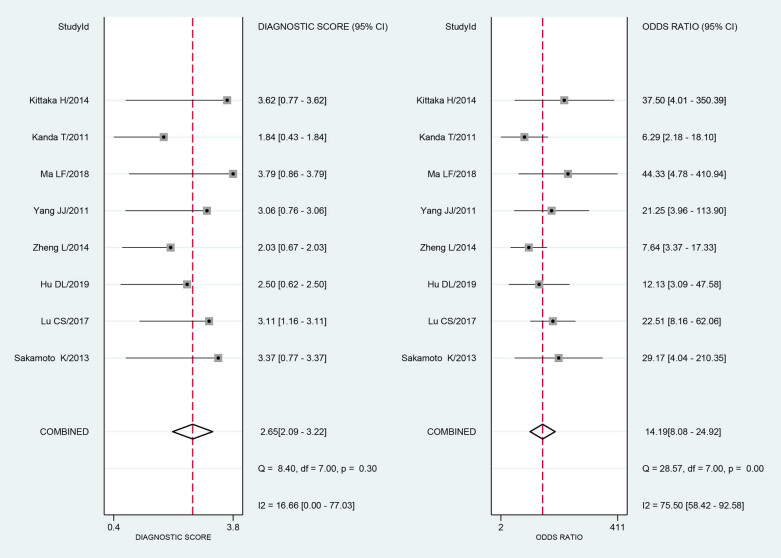
The forest plot of the DOR for serum I-FABP in diagnosis of strangulated intestinal obstruction.

The Q test and the I-square test were used to analyze the DOR, and the results were significant (*I*² = 75.50, *P* = 0.00). The meta-analysis showed that the DOR was 14.19 (95% CI: 8.08–24.92) based on the bivariate mixed-effects model (Figure 4).

### 3.5 Pooled ROC and AUC

**Figure 5:**
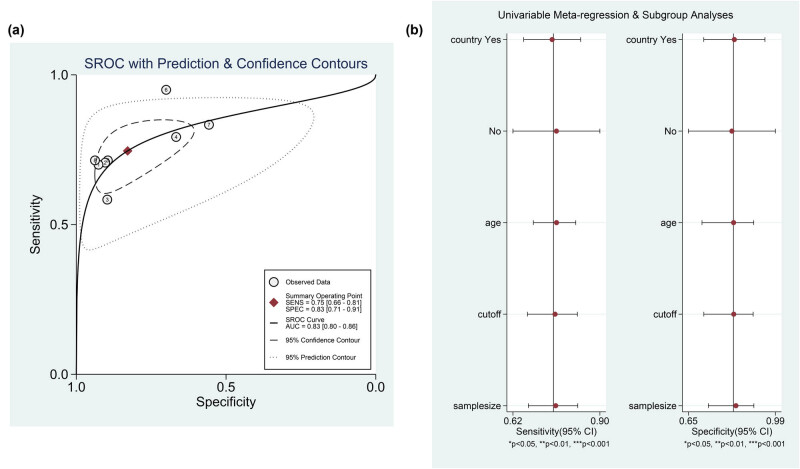
Serum I-FABP for the diagnosis of strangulated intestinal obstruction. A. ROC curve. B. Meta-regression.

Stata 15.1 was used to draw the ROC curve, and the AUC was 0.83. The accuracy of I-FABP for the diagnosis of strangulated intestinal obstruction was 0.83 (Figure 5a).

### 3.6 Analysis of the causes of heterogeneity in the included studies

The Spearman correlation coefficient (0.703, *P* = 0.078) indicated that there was no threshold effect. Further meta-regression analysis based on continuous variables such as the cutoff value, average age, sample size, and a binary covariate (country of publication) indicated that these factors were not the sources of heterogeneity (Figure 5b and Table 3).

**Table 3:** Meta-regression results

	Number of studies	Sensitivity (95% CI)	Specificity (95% CI)	*I* ^2^	*P* value
Average age	8	0.76 (0.68–0.82)	0.83 (0.71–0.91)	9	0.34
Cutoff value	8	0.75 (0.66–0.82)	0.83 (0.71–0.91)	0	0.53
Sample size	8	0.75 (0.67–0.82)	0.84 (0.73–0.91)	15	0.31
**Country**					
Japan	3	0.76 (0.62–0.90)	0.82 (0.65–0.99)	0	0.99
China	5	0.74 (0.65–0.83)	0.83 (0.71–0.95)		

### 3.7 Sensitivity analysis and publication bias

**Figure 6:**
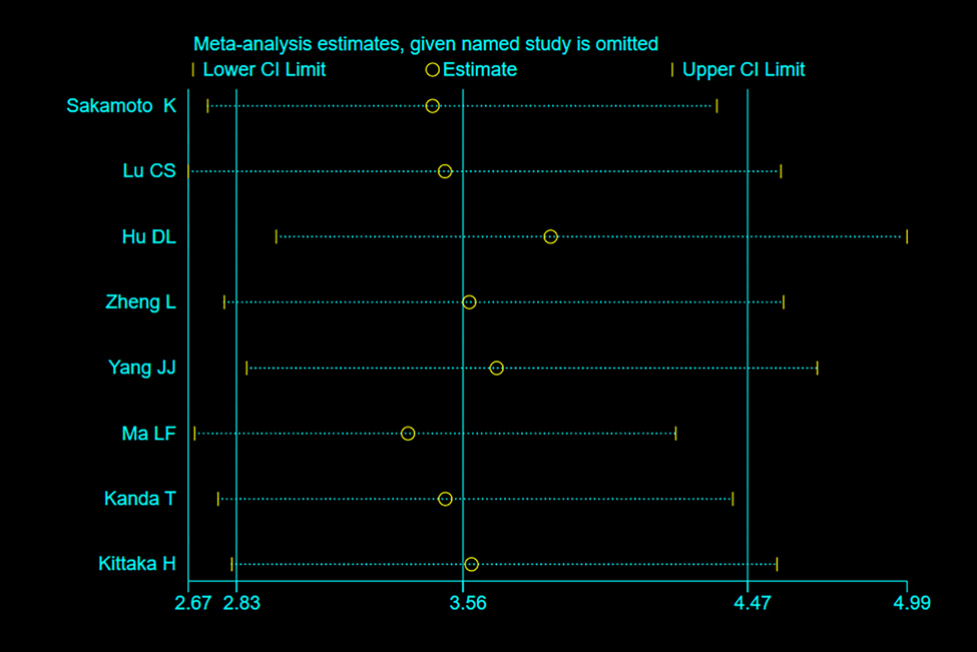
Sensitivity analysis of serum I-FABP for the diagnosis of strangulated intestinal obstruction.

**Figure 7:**
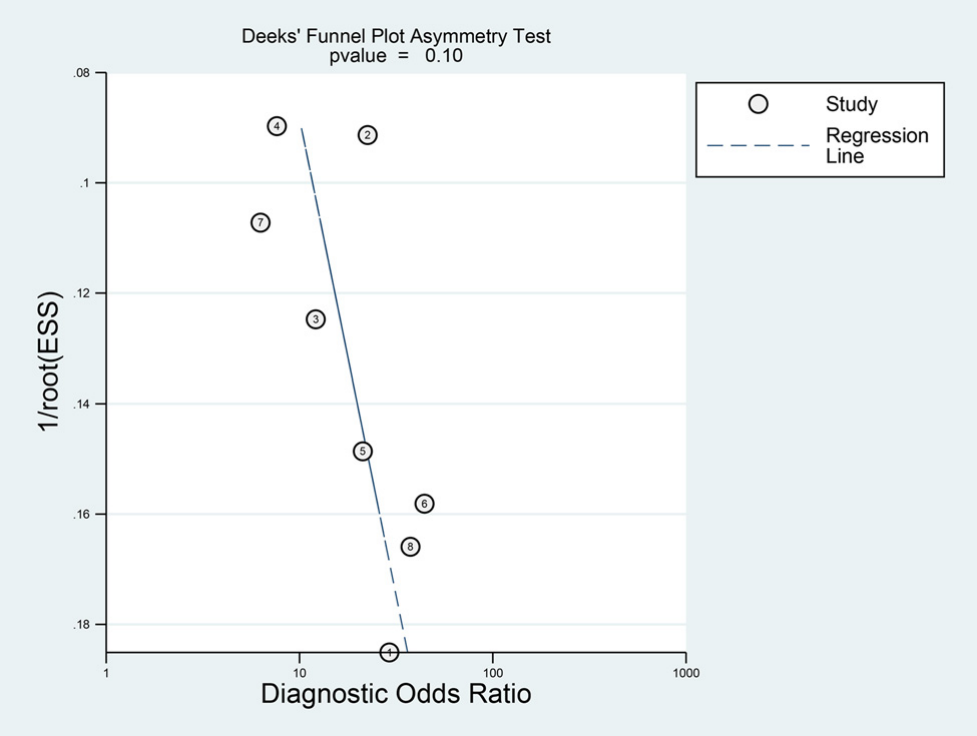
Publication bias evaluated by Deeks funnel plot.

Figure 6 shows that the deletion of each of the eight included studies did not affect the results, indicating that the results of the meta-analysis were robust. The results of the Deeks’ funnel plot asymmetry test (Figure 7) showed that *P* = 0.10, indicating that there was no publication bias.

## 4 Discussion

Strangulated intestinal obstruction is so dangerous that necessitates surgical treatment [22]. Although histopathology is the reference standard for the early diagnosis of strangulated intestinal obstruction, the slower result is substantial limitation of this method. Therefore, it is very important to explore a better method for the early diagnosis of strangulated intestinal obstruction. Some recent studies suggested that imaging, such as high-resolution CT [23], could improve the diagnosis of strangulated intestinal obstruction, but high-resolution CT is a radiological examination that is not suitable for pregnant women or children [24,25]. Moreover, high-resolution CT is expensive and cannot be used to observe the dynamic changes in patient’s condition. The detection of serum I-FABP, a unique cytoplasmic protein found in the intestinal mucosa, has the characteristics of intestinal specificity, rapid results, and simple operation. Consequently, it has attracted a great deal of attention from researchers with regard to the early diagnosis of strangulated intestinal obstruction.

A total of eight studies were included in this quantitative analysis. The meta-analysis of I-FABP for the diagnosis of strangulated intestinal obstruction showed that the pooled sensitivity was 0.75, the pooled specificity was 0.83, and the missed diagnosis rate was 25%, which is relatively high. The pooled PLR was 4.35, indicating that the I-FABP level in patients with strangulated intestinal obstruction may be 4.35 times that in patients with simple intestinal obstruction. The pooled NLR was 0.31, which suggested that 31% of the patients with intestinal obstruction with low I-FABP levels were strangulated intestinal obstruction. It may be that with the further development of strangulated intestinal obstruction to intestinal necrosis, the amount of normal intestinal villi tissue is reduced, leading to a decrease in the level of I-FABP [26]. The AUC was 0.83, indicating that the accuracy of I-FABP for the diagnosis of strangulated intestinal obstruction was 83% and suggesting that I-FABP has high diagnostic value for strangulated intestinal obstruction.

The meta-analysis showed that there was a high degree of heterogeneity among the included studies. Spearman correlation analysis suggested that there was no threshold effect; therefore, this heterogeneity originated from nonthreshold effects. There are four possible explanations for the heterogeneity. First, there were differences in the severity of the condition of the patients and the timing of the collection of blood samples, some of which were collected 15 min after admission, whereas others were collected within 24 h of admission or even after surgical confirmation of the group allocation. These differences could have resulted in selection and time interval biases. Second, differences between laboratories in different hospitals, such as the types of instruments, kits and antibodies used for the enzyme-linked immunosorbent assay (ELISA), could have caused bias. Third, the sample size of most articles in the included studies was less than 100, which might have resulted in the overestimation or underestimation of the diagnostic value of I-FABP for strangulated intestinal obstruction. However, the sample size after meta-regression analysis was not the source of heterogeneity. Hence, high-quality studies with large sample size are needed for verification in the future. Fourth, Kanda et al. [14] mentioned that the level of I-FABP increases during reversible ischemia, which could result in the overestimation of the sensitivity and the underestimation of the specificity.

Limitations of this study are as follows. First, there was heterogeneity among the included studies. Second, the basis of the diagnosis differed. Generally, strangulated intestinal obstruction is diagnosed during surgery, and simple intestinal obstruction is diagnosed based on imaging and clinical evaluation. Third, it was not clear which antibody and enzyme-linked markers were used for the ELISA in some studies. Fourth, after the application of the study inclusion and exclusion criteria, only studies published by Chinese and Japanese researchers met the criteria. Fifth, the sample size of this study was small.

In recent years, some studies [27,28,29,30,31] have shown that ischemia-modified albumin, citrulline, and smooth muscle actin (SM22) levels can be used to diagnose strangulated intestinal obstruction. The binding site of the amino terminus of albumin changes due to ischemia, reducing its ability to bind metals. This type of albumin is called ischemia-modified albumin. Montagnana et al. [1] suggested that ischemia-modified albumin may be the best predictor of ischemia and may be a sensitive biomarker of intestinal obstruction. Citrulline is only synthesized in large quantities in the intestine through the ornithine-urea pathway, and it is metabolized in the kidneys, so the organ specificity of citrulline is strong. Citrulline synthesis decreases when intestinal obstruction is strangulated. Because the muscle layer of the intestinal wall is rich in SM22, the level of SM22 increases rapidly after 4 h of intestinal wall ischemia, indicating that ischemia has reached the muscle layer. This biomarker can be used to predict the severity of strangulated intestinal obstruction and the duration of ischemia. The combination of the aforementioned biomarkers with I-FABP might increase the TP rate and reduce the rates of missed diagnosis and misdiagnosis.

In summary, I-FABP is very useful for the identification of strangulated intestinal obstruction. More standardized large-sample and high-quality studies are needed to confirm these findings.

## Abbreviations

AUC: Area under the curve
DOR: Diagnostic odds ratio
ELISA: Enzyme-linked immunosorbent assay
FP: False positive
FN: False negative
I-FABP: Intestinal fatty acid-binding protein
NLR: Negative likelihood ratio
PLR: Positive likelihood ratio
QUADAS-2: Quality assessment of diagnostic accuracy studies 2
ROC: Receiver operating characteristic
SM22: Smooth muscle actin
TP: True positive
TN: True negative
